# Time trend of mediastinal lymph node dissection in stage IA non-small cell lung cancer patient who undergo lobectomy: a retrospective study of surveillance, epidemiology, and end results (SEER) database

**DOI:** 10.1186/s13019-020-01215-x

**Published:** 2020-08-01

**Authors:** Liang Pan, Ran Mo, Linhai Zhu, Wenfeng Yu, Wang Lv, Jian Hu

**Affiliations:** Department of thoracic surgery, The First Affiliated Hospital, School of Medicine, Zhejiang University, 79 Qingchun Road, Hangzhou, 310003 China

**Keywords:** Lobectomy, Mediastinal lymph node dissection, Time trend, NSCLC

## Abstract

**Background:**

Although lobectomy with mediastinal lymph node dissection (MLND) is the first option for early-stage non-small cell lung cancer (NSCLC) patients, the time trends of MLND in stage IA NSCLC patients who undergo a lobectomy are not clear still.

**Methods:**

We included stage IA NSCLC patients who underwent lobectomy or lobectomy with MLND between 2003 and 2013 in the SEER database. The time trend of MLND was compared among patients who underwent a lobectomy.

**Results:**

For stage T1a patients, the lobectomy group and lobectomy with MLND group had no differences in postoperative overall survival (OS) (*P* = 0.34) or lung-cancer specific survival (LCSS) (*P* = 0.18) between 2003 and 2013. For stage T1b patients, the OS (*P* = 0.01) and LCSS (*P* = 0.01) were different between the lobectomy group and the lobectomy with MLND group in the period from 2003 to 2009; however, only OS (*P* = 0.04), not LCSS (*P* = 0.14), was different between the lobectomy group and the lobectomy with MLND group between 2009 and 2013. For T1c patients, the OS (*P* = 0.01) and LCSS (*P* = 0.02) were different between the two groups between 2003 and 2009 but not between 2009 and 2013 (*P* = 0.60; *P* = 0.39). From the Cox regression analysis, we found that the factors affecting OS/LCSS in T1b and T1c patients were age, sex, year of diagnosis, histology, and grade, in which year of diagnosis was the obvious factor (HR = 0.79, CI = 0.71–0.87; HR = 0.73, CI = 0.64–0.84).

**Conclusions:**

There was a time trend in prognosis differences between the lobectomy group and lobectomy with MLND group for T1b and T1c stage NSCLC patients.

## Introduction

Currently, lung cancer still has the highest incidence among malignant tumours, and NSCLC is the most common type. Although the mortality rate of lung cancer has decreased compared with the past, the five-year survival rate is still not high [1, 2]. In China, lung cancer has also long been the leading cause of cancer-related death, and there are a large number of new cases every year [3, 4]. Furthermore, with the promotion of low-dose computed tomography scans and the public’s attention to routine health examinations, more and more patients with early-stage NSCLC have emerged in clinical practice [5]. Improving the therapeutic effect for these patients has great significance for improving the survival rate of lung cancer.

For patients with early-stage NSCLC, especially with stage IA disease, surgical resection has always been the primary choice [6, 7]. However, the management of mediastinal lymph nodes during surgery remains controversial. Although early-stage NSCLC patients have been recommended for lobectomy with MLND for years, recent studies have shown that the benefit of other lymph node treatments is not inferior to MLND [8–10]. It is not clear whether MLND should be performed in stage IA NSCLC patients who undergo lobectomy [10–12].

In addition, with the development of improved treatment modalities and techniques, the impact of treatment on the prognosis of NSCLC patients has changed [13, 14], but little is known about the time trend of surgery in NSCLC patients still. In the past, most researchers focused on the surgical resection of the lung and the management of lymph nodes in early-stage NSCLC patients [15, 16], but they paid less attention to the time trend effect of surgery in early-stage patients, which is important for revealing NSCLC treatment progress. In particular, in the past decade, minimally invasive techniques and targeted therapy and immunotherapy for tumours have had a significant impact on the therapeutic effects for NSCLC patients. In early-stage NSCLC patients, this time trend effect should be considered.

The International Association for the Study of Lung Cancer (IASLC) released the eighth edition of the tumour, node, and metastasis (TNM) classification of lung cancer in 2016, and one of the changes is that the T1 stage is subdivided into T1a, T1b, and T1c [17]. In addition, the surgical outcomes of patients with NSCLC also change over time [18, 19]. However, there is little knowledge about the differences in surgical treatment and time trends among these new early-stage NSCLC patients. In this study, we compare the clinical efficacy of lobectomy and lobectomy with MLND in stage T1a, T1b and T1c NSCLC patients and time trends based on patients in the SEER database.

## Materials and methods

### Study population

This study included patients who were diagnosed with stage IA (T1a/1b/1cN0M0) NSCLC from 2003 to 2013 and had integrated clinical data in the SEER database. The patients underwent either lobectomy or lobectomy with MLND. The histological type of lung-cancer was confined to squamous cell carcinoma and adenocarcinoma (SEER codes 8170 and 8140). TNM classification of NSCLC was according to the eighth edition of the IASLC International Staging Project [17]. The exclusion criteria were as follows: 1) more than one primary tumour or coexisting multiple tumours; and 2) primary tumour sites in the main bronchus, overlapping lung lesion, and unknown sites.

Survival time was defined as the period between the date of diagnosis and the day of death. OS and LCSS were used as the main outcome events. If patients were still alive at the study cut-off date, they were regarded as censored cases.

### Statistical analysis

Categorical covariance and continuous covariance were analysed by the chi-square test and independent sample t test, respectively. The Kaplan–Meier method was utilized to show the OS distribution and LCSS distribution. A Log-rank test was used to test for significant differences between the two groups. We used the Cox proportional hazards model to perform univariate and multivariate analyses. Predictors (*P* < 0.15) identified in univariate analyses were entered into a multivariable analysis. All the data were analysed using SPSS 19.0 software (SPSS Inc., Chicago, USA) and Graph Pad Prism 5 (Graph Pad Software Inc., La Jolla, USA). *P* < 0.05 was considered statistically significant.

## Results

In this study, we enrolled 8631 stage IA NSCLC patients who underwent lobectomy or lobectomy with MLND between 2003 and 2013 totally and grouped these patients into the lobectomy group and lobectomy with MLND group. Based on tumour size and time of diagnosis, these patients were divided into six groups for stratified studies. The baseline characteristics of stage IA (T1a, T1b, and T1c) NSCLC patients who underwent surgery between 2003 and 2013 are listed in Table 1, Table 2 and Table 3. Perhaps due to patient stratification, there were almost no significant differences in these preoperative variables between the lobectomy group and the lobectomy with MLND group, except for the primary site of the tumour in Table 2 and Table 3.

**Table 1:** Baseline characteristics of T1a NSCLC patients who were diagnosed in 2003–2013

Variables	Period between 2003 and 2008			Period between 2009 and 2013		
	Lobectomy (*n* = 98)	Lobectomy with MLND (*n* = 215)	*p*	Lobectomy (*n* = 82)	Lobectomy with MLND (*n* = 295)	*p*
Mean ± SD Age (years)	63.55 ± 9.56	65.53 ± 8.83	0.08	64.51 ± 10.45	64.65 ± 9.08	0.91
Sex, no. (%)			0.09			0.15
male	46 (46.9)	79 (36.7)		36 (43.9)	104 (35.3)	
female	52 (53.1)	136 (63.3)		46 (56.1)	191 (64.7)	
Race, no. (%)			0.90			0.89
White	88 (89.8)	192 (89.3)		67 (81.7)	243 (82.4)	
Black/ Others	10 (10.2)	23 (10.7)		15 (18.3)	52 (17.6)	
Histology, no. (%)			0.67			0.22
Squamous cell carcinoma	30 (30.6)	71 (33.0)		15 (18.3)	73 (24.7)	
Adenocarcinoma	68 (69.4)	144 (67.0)		67 (81.7)	222 (75.3)	
Grade, no. (%)			0.18			0.71
I	16 (16.3)	42 (19.5)		24 (29.3)	91 (30.8)	
II	59 (60.2)	99 (46)		34 (41.5)	135 (45.8)	
III	19 (19.4)	59 (27.4)		22 (26.8)	59 (20.0)	
IV	0	1 (0.5)		0	1 (0.3)	
Unknown	4 (4.1)	14 (6.5)		2 (2.4)	9 (3.1)	
Primary site of tumor, no. (%)			0.86			0.71
Upper lobe	70 (71.4)	147 (68.4)		51 (62.2)	192 (65.1)	
Middle lobe	6 (6.1)	15 (7.0)		8 (9.8)	21 (7.1)	
Lower lobe	22 (22.4)	53 (24.7)		23 (28)	82 (27.8)	
Laterality, no. (%)			0.31			0.94
Left	39 (39.8)	72 (33.5)		29 (35.4)	103 (34.9)	
Right	59 (60.2)	143 (66.5)		53 (64.6)	192 (65.1)	

*SD* Standard deviation

T1a 0 < tumor size≤1 cm

**Table 2:** Baseline characteristics of T1b NSCLC patients who were diagnosed in 2003–2013

Variables	Period between 2003 and 2008			Period between 2009 and 2013		
	Lobectomy (*n* = 703)	Lobectomy with MLND (*n* = 1333)	*p*	Lobectomy (*n* = 484)	Lobectomy with MLND (*n* = 1838)	*p*
Mean ± SD Age (years)	66.94 ± 9.66	66.10 ± 9.71	0.06	66.98 ± 9.29	66.48 ± 9.23	0.28
Sex, no. (%)			0.39			0.24
male	312 (44.4)	565 (42.4)		222 (45.9)	788 (42.9)	
female	391 (55.6)	768 (57.6)		262 (54.1)	1050 (57.1)	
Race, no. (%)			0.99			0.70
White	613 (87.2)	1162 (87.2)		408 (84.3)	1536 (83.6)	
Black/ Others	90 (12.8)	171 (12.8)		76 (15.7)	302 (16.4)	
Histology, no. (%)			0.18			0.46
Squamous cell carcinoma	215 (30.6)	370 (27.8)		135 (27.9)	482 (26.2)	
Adenocarcinoma	488 (69.4)	963 (72.2)		349 (72.1)	1356 (73.8)	
Grade, no. (%)			0.62			0.47
I	103 (14.7)	177 (13.3)		85 (17.6)	370 (20.1)	
II	363 (51.6)	702 (52.7)		251 (51.9)	971 (52.8)	
III	210 (29.9)	417 (31.3)		130 (26.9)	444 (24.2)	
IV Unknown	3 (0.4)	4 (0.3)		2 (0.4)	4 (0.2)	
	24 (3.4)	33 (2.5)		16 (3.3)	49 (2.7)	
Primary site of tumor, no. (%)			0.01			0.31
Upper lobe	463 (65.9)	890 (66.8)		308 (63.6)	1198 (65.2)	
Middle lobe	62 (8.8)	66 (5.0)		33 (6.8)	93 (5.1)	
Lower lobe	178 (25.3)	377 (28.3)		143 (29.5)	547 (29.8)	
Laterality, no. (%)			0.50			0.38
Left	283 (40.3)	516 (38.7)		195 (40.3)	700 (38.1)	
Right	420 (59.7)	817 (61.3)		289 (59.7)	1138 (61.9)	

*SD* Standard deviation

T1b 1 cm < tumor size≤2 cm

**Table 3:** Baseline characteristics of T1c NSCLC patients who were diagnosed in 2003–2013

Variables	Period between 2003 and 2008			Period between 2009 and 2013		
	Lobectomy (*n* = 563)	Lobectomy with MLND (*n* = 1158)	*p*	Lobectomy (*n* = 363)	Lobectomy with MLND (*n* = 1499)	*p*
Mean ± SD Age (years)	67.60 ± 9.76	68.31 ± 9.32	0.14	67.94 ± 9.55	68.13 ± 9.06	0.71
Sex, no. (%)			0.14			0.82
male	295 (52.4)	563 (48.6)		169 (46.6)	708 (47.2)	
female	268 (47.6)	595 (51.4)		194 (53.4)	791 (52.8)	
Race, no. (%)			0.93			0.49
White	489 (86.9)	1004 (86.7)		297 (81.8)	1249 (83.3)	
Black/ Others	74 (13.1)	154 (13.3)		66 (18.2)	250 (16.7)	
Histology, no. (%)			0.82			0.62
Squamous cell carcinoma Adenocarcinoma	199 (35.3)	416 (35.9)		127 (35.0)	504 (33.6)	
	364 (64.7)	742 (64.1)		236 (65.0)	995 (66.4)	
Grade, no. (%)			0.44			0.86
I	54 (9.6)	131 (11.3)		52 (14.3)	239 (15.9)	
II	274 (48.7)	554 (47.8)		186 (51.2)	749 (50.0)	
III	216 (38.4)	434 (37.5)		117 (32.2)	467 (31.2)	
IV	2 (0.4)	11 (0.9)		1 (0.3)	6 (0.4)	
Unknown	17 (3.0)	28 (2.4)		7 (1.9)	38 (2.5)	
Primary site of tumor, no. (%)			0.01			0.07
Upper lobe	353 (62.7)	764 (66.0)		214 (59.0)	978 (65.2)	
Middle lobe	37 (6.6)	40 (3.5)		18 (5.0)	55 (3.7)	
Lower lobe	173 (30.7)	354 (30.6)		131 (35.1)	466 (31.1)	
Laterality, no. (%)			0.11			0.54
Left	219 (38.9)	498 (43.0)		159 (43.8)	630 (42.0)	
Right	344 (61.1)	660 (57.0)		204 (56.2)	869 (58.0)	

*SD* Standard deviation

T1c 2 < tumor size≤3 cm

**Fig. 1:**
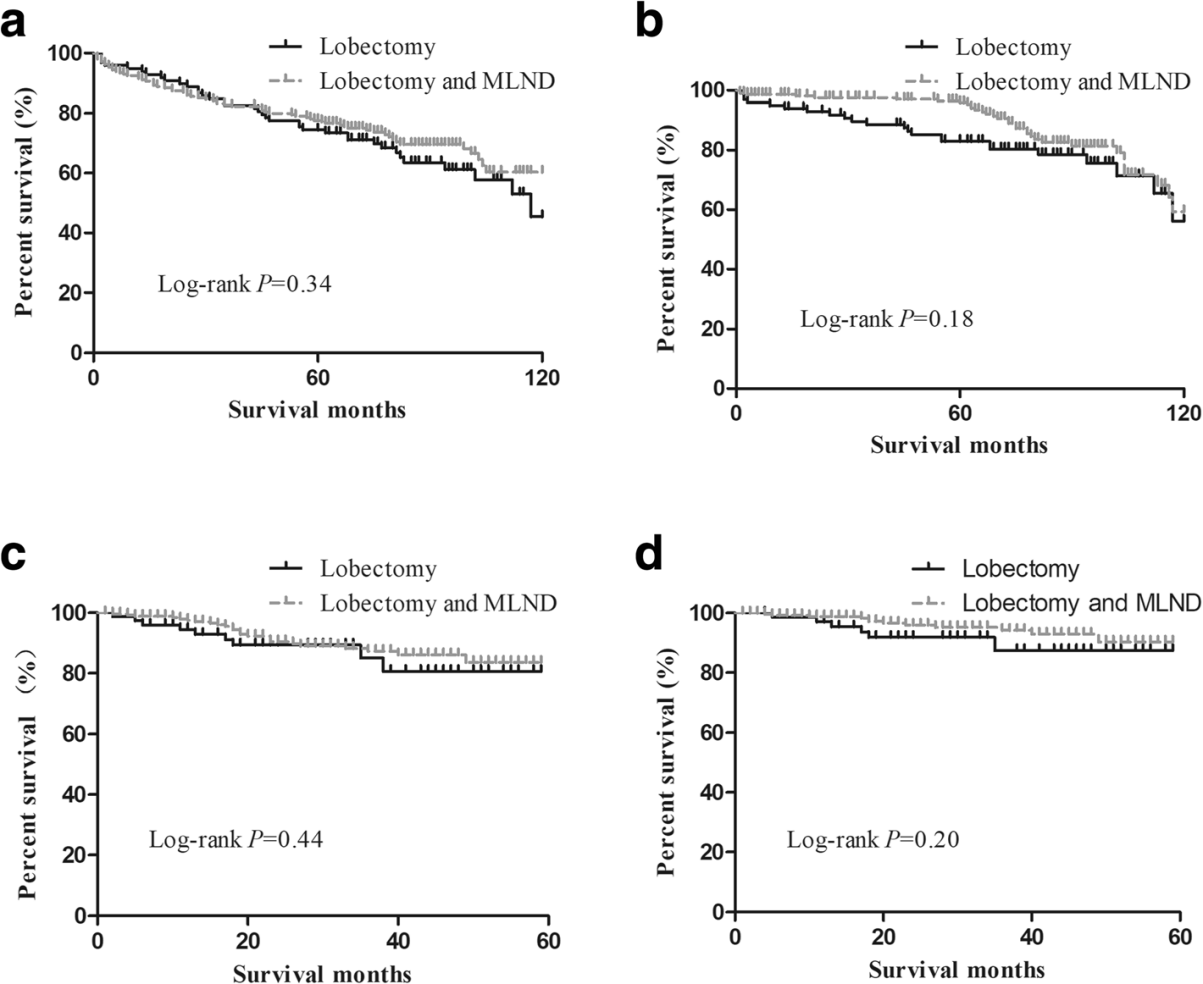
Kaplan–Meier survival curve in T1a patient groups (**a**) OS curve in period 2003–2008, (**b**) LCSS curve in period 2003–2008, (**c**) OS curve in period 2009–2013, and (**d**) LCSS curve in period 2009–2013

**Fig. 2:**
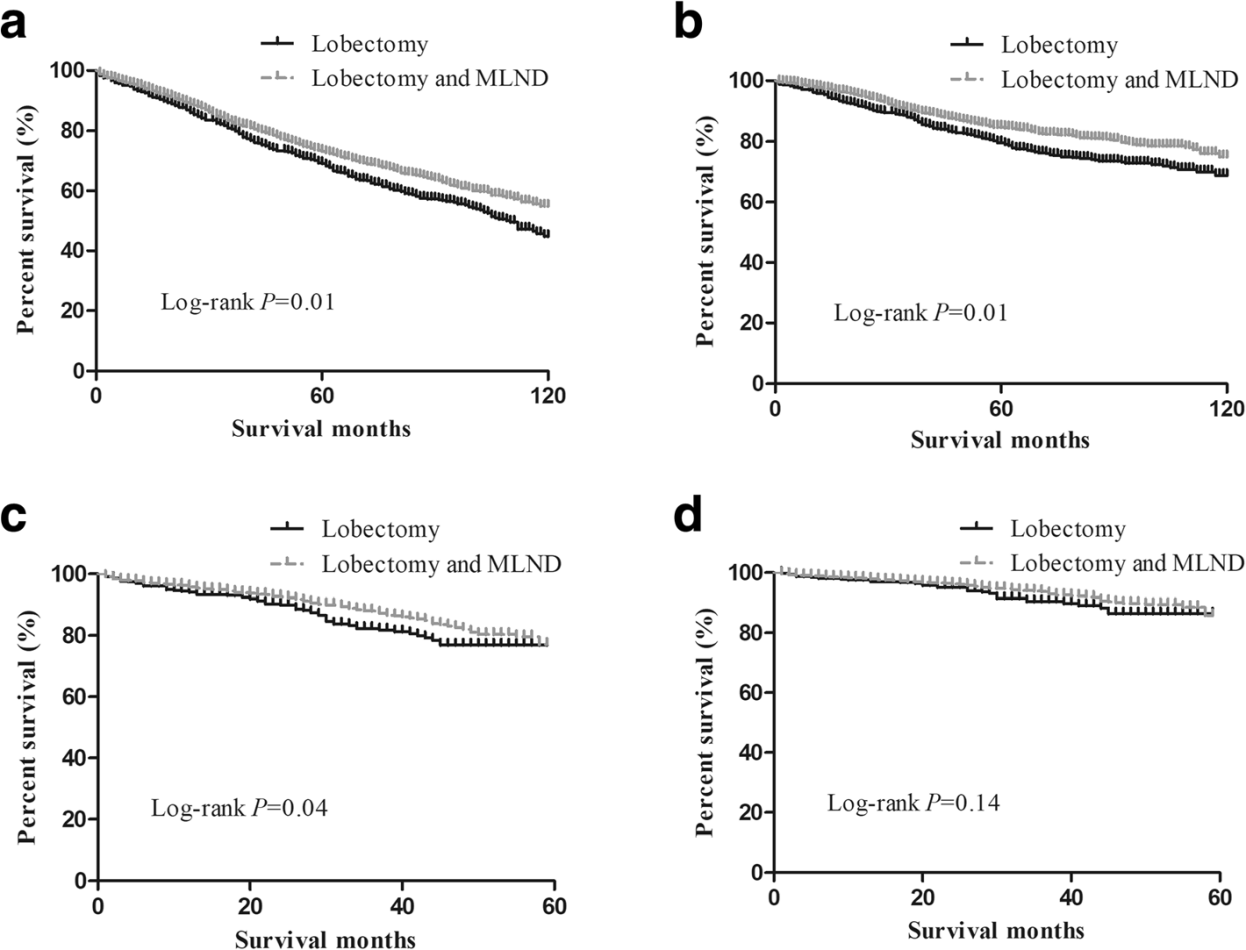
Kaplan–Meier survival curve in T1b patient groups (**a**) OS curve in period 2003–2008, (**b**) LCSS curve in period 2003–2008, (**c**) OS curve in period 2009–2013, and (**d**) LCSS curve in period 2009–2013

**Fig. 3:**
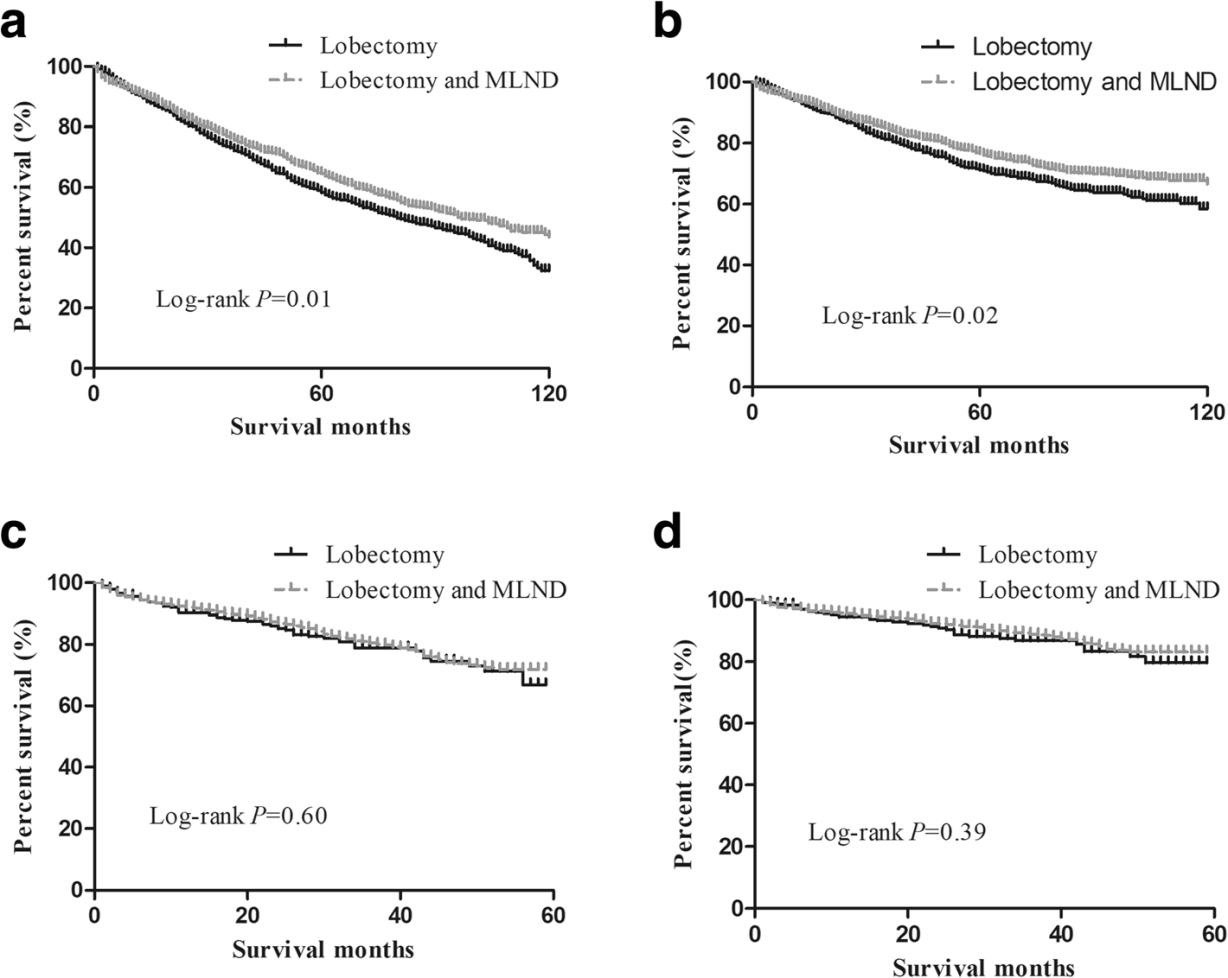
Kaplan–Meier survival curve in T1c patient groups (**a**) OS curve in period 2003–2008, (**b**) LCSS curve in period 2003–2008, (**c**) OS curve in period 2009–2013, and (**d**) LCSS curve in period 2009–2013

Then, we plotted the overall survival curve and lung cancer-specific survival curve of the groups and performed a log-rank test (Figs. 1, 2, 3). For T1a NSCLC patients, in both the period between 2003 and 2008 (Fig. 1 a, b) and the period between 2009 and 2013 (Fig. 1 c, d), there was no significant difference in the OS (log-rank *p* = 0.34, *p* = 0.44) and LCSS (log-rank *p* = 0.18, *p* = 0.20) between the lobectomy group and the lobectomy with MLND group. However, for stage T1b and T1c patients, the survival status was different. Between 2003 and 2008, the survival statuses of the lobectomy group and lobectomy with MLND group was significantly different in T1b patients, in terms of both OS (Fig. 2 a, log-rank *p* = 0.01) and LCSS (Fig. 2 b, log-rank *p* = 0.01). Nevertheless, we found that the lobectomy group and lobectomy with MLND group had different OS (Fig. 2 c, log-rank *p* = 0.04), but not LCSS (Fig. 2 d, log-rank *p* = 0.14), in T1b patients between 2009 and 2013. For T1c patients, we also found something different. The OS and LCSS were significantly different between 2003 and 2008 (Fig. 3 a, b log-rank *p* = 0.01, log-rank *p* = 0.02) between the two groups, but not between 2009 and 2013 (Fig. 3 c, d log-rank *p* = 0.60, log-rank *p* = 0.39).

To further identify prognostic factors of the OS and LCSS in T1b and T1c patients, we performed Cox regression analyses. The factors affecting the prognosis status of patients are shown in Table 4. For the OS of NSCLC patients, the factors affecting patients prognosis were age (HR = 1.04, 95% CI 1.04–1.05, *p* = 0.01), sex (HR = 0.73, 95% CI 0.67–0.79, *p* = 0.01), year of diagnosis (HR = 0.79, 95% CI 0.71–0.87, *p* = 0.01), histology (HR = 0.76, 95% CI 0.70–0.83, *p* = 0.01) and grade (HR = 1.13, 95% CI 1.08–1.19, *p* = 0.01). The factors affecting the LCSS of patients were age (HR = 1.03, 95% CI 1.02–1.04, *p* = 0.01), sex (HR = 0.76, 95% CI 0.68–0.85, *p* = 0.01), year of diagnosis (HR = 0.73, 95% CI 0.64–0.84, *p* = 0.01),and grade (HR = 1.20, 95% CI 1.12–1.28, *p* = 0.01).

**Table 4:** Cox regression analyses for OS and LCSS in T1b and T1c NCSLC patients who underwent Lobectomy or Lobectomy with MLND between 2003 and 2013

variables	OS				LCSS			
	p	Exp(B)	95.0% CI for Exp(B)		P	Exp(B)	95.0% CI for Exp(B)	
			Lower	Upper			Lower	Upper
Age	0.01	1.042	1.037	1.047	0.01	1.03	1.02	1.04
Sex	0.01	0.729	0.671	0.793	0.01	0.76	0.68	0.85
Year of diagnosis	0.01	0.786	0.709	0.871	0.01	0.73	0.64	0.84
Histology	0.01	0.758	0.695	0.828	–	–	–	–
Grade	0.01	1.132	1.076	1.191	0.01	1.20	1.12	1.28

OS overall survival LCSS lung cancer specific survival

HR hazard ratio CI confidence interval

## Discussion

The treatment of mediastinal lymph nodes in NSCLC patients has always been controversial, especially in patients with early-stage NSCLC. The European Society of Thoracic Surgeons (ESTS) guidelines recommend the sampling or dissection of systemic lymph nodes in all lung cancer patients, but the National Comprehensive Cancer Network (NCCN) and the American College of Chest Physicians (ACCP) did not exclude other treatments of mediastinal lymph nodes in NSCLC patients [20–22]. In this study, our results indicated that as time progresses and treatments advance, the survival rate of stage IA NSCLC patients who underwent lobectomy was not inferior to patients who underwent lobectomy with MLND. In particular, the benefit of lobectomy in T1a NSCLC patients has always been noninferior to lobectomy with MLND, but this is not the case in T1b and T1c NSCLC patients. Therefore, stage IA NSCLC patients may undergo lobectomy for surgical resection. Of course, this needs to be confirmed by larger prospective randomized controlled studies in the future.

Darling GE et al. showed that for N0 NSCLC patients with negative results from the systematic sampling of mediastinal lymph nodes, MLND does not improve survival in these patients with early-stage NSCLC patients [10]. Hiroyuki et al. also indicated that the OS and LCSS of lobe-specific nodal dissection were roughly equivalent to those of MLND in early-stage NSCLC patients [10]. These conclusions are consistent with our findings. However, some studies have also noted that there is a phenomenon of skipping metastasis in lymph node metastasis of NSCLC, even in stage IA patients, and the mediastinal lymph node dissection can obtain accurate staging and provide guidance for postoperative chemotherapy and radiotherapy [23–25]. Indeed, we do not deny the superiority of mediastinal lymph node dissection in N stage patients with NSCLC, but the incidence of skipping metastasis in patients with stage IA NSCLC is not high. In addition, as an increasing number of targeted therapies achieve very good clinical results, these methods are a good complement to the inaccuracies of N stage in surgery. Considering the potential for injuring of the recurrent laryngeal nerve and esophagus, and possible complications caused by MLND, lobectomy with MLND is not the most appropriate choice for all patients with stage IA NSCLC.

In addition, there were also researchers hold the opinion that for potentially better survival, patients who are intraoperatively identified as stage T1 with lesions between 2 and 3 cm should undergo systematic MLND, and patients with lesions of 2 cm or less should undergo mediastinal lymph node sampling [26, 27]. However, the significant differences between this study and the study above are that the sample size and study time. Therefore, we took advantage of the SEER database and conducted a large-scale retrospective study to compare the time trend between the lobectomy group and lobectomy with the MLND group. We found that lobectomy with MLND is not superior in terms of postoperative survival compared to lobectomy in stage T1 NSCLC patients with lesions between 2 and 3 cm or with fewer lesions. Certainly, this finding was due to many factors. For example, with the advancement of surgical techniques, such as video-assisted thoracoscopic surgery (VATS) and Da Vinci surgical robot, lobectomy has become increasingly minimally invasive and rapid [28–30]. However, MLND may inevitably cause damage to the oesphagus, recurrent laryngeal nerve and other important tissues. In addition, with the rise of targeted therapy and immunotherapy, more and more advanced NSCLC patients benefit from these treatments, and to some extent, new treatments may compensate for the inaccuracy of surgical clinical staging. As the results show, the LCSS of T1b and T1c NSCLC patients differed between the lobectomy group and lobectomy with MLND group in the period 2003–2008. However, there was no difference between the two groups in the period from 2009 to 2013. This is a reflection of the time trends. To the best of our knowledge, this is the first time a study has evaluated the impact of mediastinal lymph node dissection on the prognosis of T1a, T1b, and T1c NSCLC patients undergoing lobectomy respectively according to the eighth edition of the lung cancer staging criteria.

This study also had some limitations. For example, the SEER database did not have pathological data regarding the postoperative N stage, so we were unable to determine the effect of the two surgical procedures on the postoperative N stage. In addition, there are no preoperative and postoperative chemotherapy data in the database, which will have a certainly impact the judgment of the results. Moreover, although this is a large-scale data study, it is a retrospective study, and the results are not as convincing as large-scale prospective multicentre studies.

In conclusion, our results suggest that for stage IA NSCLC patients, lobectomy was not inferior to lobectomy with MLND in T1 NSCLC patients. Moreover, the opposite result was found in the study periods between 2003 and 2008 and study period between 2009 and 2013 for T1b and T1c NCSLC patients, which means that there is a time trend of the effect that two surgical procedures have on these patients that we should pay attention to.

## Data Availability

The datasets used and/or analysed during the current study are available from the SEER database (https://seer.cancer.gov/data/).

## References

[1] Visbal AL, Williams BA, Nichols FC (2004). Gender differences in non-small-cell lung cancer survival: an analysis of 4,618 patients diagnosed between 1997 and 2002. Ann Thorac Surg.

[2] Jemal A, Siegel R, Ward E (2008). Cancer statistics, 2008. CA Cancer J Clin.

[3] Chen W, Zheng R, Baade PD (2016). Cancer statistics in China, 2015. CA Cancer J Clin.

[4] Chen W, Zheng R, Zeng H (2015). Epidemiology of lung cancer in China. Thorac Cancer.

[5] Moyer VA (2014). Screening for lung cancer: U.S. preventive services task force recommendation statement. Ann Intern Med.

[6] Scott WJ, Howington J, Feigenberg S (2007). Treatment of non-small cell lung cancer stage I and stage II: ACCP evidence-based clinical practice guidelines (2nd edition). Chest.

[7] Monirul Islam KM, Shostrom V, Kessinger A (2013). Outcomes following surgical treatment compared to radiation for stage I NSCLC: a SEER database analysis. Lung Cancer.

[8] Wu Y, Huang ZF, Wang SY (2002). A randomized trial of systematic nodal dissection in resectable non-small cell lung cancer. Lung Cancer.

[9] Darling GE, Allen MS, Decker PA (2011). Randomized trial of mediastinal lymph node sampling versus complete lymphadenectomy during pulmonary resection in the patient with N0 or N1 (less than hilar) non-small cell carcinoma: results of the American college of surgery oncology group Z0030 trial. J Thorac Cardiovasc Surg.

[10] Adachi H, Sakamaki K, Nishii T (2017). Lobe-specific lymph node dissection as a standard procedure in surgery for non-small cell lung cancer: a propensity score matching study. J Thorac Oncol.

[11] Ou SH, Zell JA (2008). Prognostic significance of the number of lymph nodes removed at lobectomy in stage IA non-small cell lung cancer. J Thorac Oncol.

[12] Miller DL, Rowland CM, Deschamps C (2002). Surgical treatment of non-small cell lung cancer 1 cm or less in diameter. Ann Thorac Surg.

[13] Reck M, Rabe KF (2017). Precision diagnosis and treatment for advanced non-small-cell lung cancer. N Engl J Med.

[14] Herbst RS, Morgensztern D, Boshoff C (2018). The biology and management of non-small cell lung cancer. Nature.

[15] Altorki NK, Wang X, Wigle D (2018). Perioperative mortality and morbidity after sublobar versus lobar resection for early-stage non-small-cell lung cancer: post-hoc analysis of an international, randomised, phase 3 trial (CALGB/Alliance 140503). Lancet Respir Med.

[16] Dai C, Shen J, Ren Y (2016). Choice of surgical procedure for patients with non-small-cell lung cancer </= 1 cm or > 1 to 2 cm among lobectomy, Segmentectomy, and wedge resection: a population-based study. J Clin Oncol.

[17] Goldstraw P, Chansky K, Crowley J (2016). The IASLC lung cancer staging project: proposals for revision of the TNM stage groupings in the forthcoming (eighth) edition of the TNM classification for lung cancer. J Thorac Oncol.

[18] Hanagiri T, Baba T, So T (2010). Time trends of surgical outcome in patients with non-small cell lung cancer. J Thorac Oncol.

[19] Yendamuri S, Sharma R, Demmy M (2013). Temporal trends in outcomes following sublobar and lobar resections for small (</= 2 cm) non-small cell lung cancers--a surveillance epidemiology end results database analysis. J Surg Res.

[20] Lardinois D, De Leyn P, Van Schil P (2006). ESTS guidelines for intraoperative lymph node staging in non-small cell lung cancer. Eur J Cardiothorac Surg.

[21] Kurzrock R, Colevas AD, Olszanski A (2015). NCCN oncology research Program's investigator steering committee and NCCN best practices committee molecular profiling surveys. J Natl Compr Canc Netw.

[22] Howington JA, Blum MG, Chang AC (2013). Treatment of stage I and II non-small cell lung cancer: diagnosis and management of lung cancer, 3rd ed: American College of Chest Physicians evidence-based clinical practice guidelines. Chest.

[23] Dai C, Xie H, Kadeer X (2017). Relationship of lymph node micrometastasis and micropapillary component and their joint influence on prognosis of patients with stage I lung adenocarcinoma. Am J Surg Pathol.

[24] Funatsu T, Matsubara Y, Ikeda S (1994). Preoperative mediastinoscopic assessment of N factors and the need for mediastinal lymph node dissection in T1 lung cancer. J Thorac Cardiovasc Surg.

[25] Prenzel KL, Baldus SE, Monig SP (2004). Skip metastasis in nonsmall cell lung carcinoma: predictive markers and isolated tumor cells in N1 lymph nodes. Cancer.

[26] Ma K, Chang D, He B (2008). Radical systematic mediastinal lymphadenectomy versus mediastinal lymph node sampling in patients with clinical stage IA and pathological stage T1 non-small cell lung cancer. J Cancer Res Clin Oncol.

[27] Sugi K, Nawata K, Fujita N (1998). Systematic lymph node dissection for clinically diagnosed peripheral non-small-cell lung cancer less than 2 cm in diameter. World J Surg.

[28] Bendixen M, Jorgensen OD, Kronborg C (2016). Postoperative pain and quality of life after lobectomy via video-assisted thoracoscopic surgery or anterolateral thoracotomy for early stage lung cancer: a randomised controlled trial. Lancet Oncol.

[29] Yan TD, Black D, Bannon PG (2009). Systematic review and meta-analysis of randomized and nonrandomized trials on safety and efficacy of video-assisted thoracic surgery lobectomy for early-stage non-small-cell lung cancer. J Clin Oncol.

[30] Cerfolio RJ, Bryant AS, Skylizard L (2011). Initial consecutive experience of completely portal robotic pulmonary resection with 4 arms. J Thorac Cardiovasc Surg.

